# Uncertainty Management in Assessment of FMEA Expert Based on Negation Information and Belief Entropy

**DOI:** 10.3390/e25050800

**Published:** 2023-05-15

**Authors:** Lei Wu, Yongchuan Tang, Liuyuan Zhang, Yubo Huang

**Affiliations:** 1 School of Information Science and Engineering, Zaozhuang University, Zaozhuang 277160, China; 2 School of Microelectronics, Northwestern Polytechnical University, Xi’an 710072, China; 3 School of Big Data and Software Engineering, Chongqing University, Chongqing 401331, China; 4 School of Engineering, University of Warwick, Coventry CV4 7AL, UK

**Keywords:** Dempster–Shafer evidence theory, uncertainty, negation evidence, belief entropy, multi-source information fusion

## Abstract

The failure mode and effects analysis (FMEA) is a commonly adopted approach in engineering failure analysis, wherein the risk priority number (RPN) is utilized to rank failure modes. However, assessments made by FMEA experts are full of uncertainty. To deal with this issue, we propose a new uncertainty management approach for the assessments given by experts based on negation information and belief entropy in the Dempster–Shafer evidence theory framework. First, the assessments of FMEA experts are modeled as basic probability assignments (BPA) in evidence theory. Next, the negation of BPA is calculated to extract more valuable information from a new perspective of uncertain information. Then, by utilizing the belief entropy, the degree of uncertainty of the negation information is measured to represent the uncertainty of different risk factors in the RPN. Finally, the new RPN value of each failure mode is calculated for the ranking of each FMEA item in risk analysis. The rationality and effectiveness of the proposed method is verified through its application in a risk analysis conducted for an aircraft turbine rotor blade.

## 1. Introduction

Uncertainty management is a key issue in many applications, especially for uncertain circumstances [1]. At present, a considerable number of methods have been proposed to deal with uncertainty by displaying information in a certain framework, such as Dempster–Shafer evidence theory (DST) [2,3], fuzzy set theory [4,5,6,7,8,9], rough sets theory [10,11], D number theory [12,13], and R number theory [14,15]. Among these frameworks, Dempster–Shafer evidence theory has undergone rapid development due to its effectiveness in addressing uncertain information [16,17]. It has been widely used in many applications, such as decision making [18,19,20], classification [21], fault diagnosis [22,23,24,25], risk evaluation [26,27,28], and so on [29,30]. However, how to handle uncertainty under the framework of DST is still an open issue [31,32].

Deng entropy was proposed to measure the uncertainty of basic probability assignments (BPA) in the framework of DST [33]. Compared with other measures, such as the ambiguity measure [34], decomposable entropy [35,36], correlation coefficient [37], and other methods for uncertainty management in DST, the Deng entropy has some advantages. For example, Deng entropy has the same calculation result as Shannon entropy if the mass function is for a single set and the BPA degenerates to a probability distribution. Therefore, the Deng entropy is adopted to measure the uncertainty of experts’ assessments in this work.

As a typical bottom-up technique for potential risk modeling and management [38], since it was introduced by NASA in 1960s [39], failure mode and effects analysis (FMEA) has been extensively used in practical applications, such as medical treatment [40,41], aircraft landing systems [42,43], the automotive industry [44,45], software engineering [46,47], and so on [48,49]. Traditional FMEA processes can be divided into five steps, including (1) assembling a team, (2) determining the scope of FMEA, (3) identifying potential failure modes and effects, (4) calculating the risk priority number (RPN) of each failure mode and ranking, and (5) reporting the analysis results [50]. However, due to the increasing complexity of systems, the processes of the FMEA approach may exhibit uncertainty in the subjective assessments of the experts. Therefore, the conventional RPN model may not always be efficient in practical applications [51,52,53].

The study of negation information brings significant implications for knowledge representation and uncertainty measurement, as the beliefs of each focal element can impact the negation of other focal elements. A more general method for determining the negation of BPA has been proposed by Yin et al. [54]. Inspired by the above methods, a novel RPN model called the negation BPA-based risk priority number with Deng entropy (nRPN) is proposed in this article to represent the relative importance of each risk factor in FMEA. In this method, the Deng entropy is used to measure the uncertainty of the evidence. The negation of BPA is calculated. Following that, the weights of three risk factors according to the FMEA experts’ assessments are calculated. Ultimately, the new RPN is calculated based on a new formula considering the aforementioned weight factor and negation information. Compared with other improved FMEA approaches, there are several features of the proposed method. First, the Deng entropy is an efficient method for obtaining the uncertainty degree of BPA. Second, the proposed model considers more uncertain information by utilizing the negation evidence, ensuring internal coordination in comparison to methods based on fuzzy set theory and so on [50,55]. Finally, the identification of failure modes becomes easier due to the significant difference between the decision values, which is an improvement over some previous methods [56].

The rest of this paper is organized as follows. The preliminaries are introduced in Section 2. Section 3 conveys a novel approach named the negation BPA-based risk priority number (nRPN) with Deng entropy for the FMEA model. In Section 4, a case study of an aircraft turbine rotor blade is analyzed in detail with the proposed method. Finally, Section 5 provides the conclusion of this paper.

## 2. Preliminaries

### 2.1. Dempster–Shafer Evidence Theory

**Definition** **1.** *The set of all observed events is represented in the frame of discernment (FOD). Let* Ω *be the non-empty set consisting of mutually exclusive and collectively exhaustive events $E_{i}$,where* Ω *is the FOD, indicated as*

(1) $$\Omega =\{E_{1},\;E_{2},\cdots ,E_{i},\cdots ,E_{n}\}$$

*The power set of* Ω *consists of $2^{n}$ elements as denoted by $2^{\Omega }$, that is,*

(2) $$2^{\Omega }=\{\emptyset ,\{E_{1}\},\cdots ,\{E_{n}\},\{E_{1},E_{2}\},\cdots ,\{E_{1},E_{2},\cdots ,E_{i}\},\cdots ,\Omega \}$$

*where the* ∅ *is an empty set.*

**Definition** **2.** *A mass function m is a mapping from $2^{\Omega }$ to the interval [0, 1], which is also called a basic probability assignment (BPA). It is used to transform the event to a probability, formally defined as:*

(3) $$m\;:2^{\Omega }\to [0,1]$$

*where m satisfies*

(4) $$m(\emptyset )=0\;and\sum_{A\in \Omega }m(A)=1,\;0\leq m(A)\leq 1$$

*here, A is called a focal element, and m(A) represents the belief to A [54].*

**Definition** **3.** *In evidence theory, the belief function Bel and plausibility function Pl can also express the mass function. The Bel and Pl are defined as:*

(5) $$Bel(A)=\sum_{\emptyset \neq B\subseteq A}m(B),\;\;Pl(A)=\sum_{B\cap A\neq \emptyset }m(B).$$

*The belief function Bel(A) represents the justified specific support of the focal element A, while the plausibility function Pl(A) represents the potential specific support [2].*

**Definition** **4.** *Assume that there are two pieces of evidence, which are indicated as $m_{1}$ and $m_{2}$. The focal elements of $m_{1}$ are represented as B, and the focal elements of $m_{2}$ are represented as C. The independent mass functions can be fused by Dempster’s combination rule [2,3], which is defined as follows:*

(6) $$m(A)=(m_{1}⊕m_{2})(A)=\frac{1}{1-k}\sum_{B\cap C=A}m_{1}(B)m_{2}(C)$$

*where the coefficient k is a normalization factor defined as follows:*

(7) $$k=\sum_{B\cap C=\emptyset }m_{1}(B)m_{2}(C)$$

### 2.2. Failure Mode and Effects Analysis

Failure mode and effects analysis (FMEA) is a widely used analytical tool for potential risk modeling and management. FMEA has been applied extensively in many fields, such as process management (PFMEA), system management (SFMEA), product design (DFMEA), and so on. A crucial step in the traditional FMEA application process involves prioritizing failure modes based on the risk priority number (RPN).

**Definition** **5.** *The risk priority number (RPN) is composed of three items: the probability of the occurrence of an FMEA item (O), the severity degree if a failure happens (S), and the probability of a potential failure being detected (D), which can be defined as*

(8) RPN=O×S×D

*Generally, there are 10 ranking levels for each risk factor, from 1 to 10 [56].*

### 2.3. Deng Entropy

The Deng entropy is an efficient method to measure the degree of uncertainty of BPA [33].

**Definition** **6.** *The Deng entropy is defined as [33]*

(9) $$E_{d}(m)=\;-\;\sum_{A\subseteq X}m(A)\mathrm{lo}g_{2}\frac{m(A)}{2^{\left|A\right|}-1}\;$$

*where m is the mass function defined in the frame of discernment X, A is a focal element, and |A| stands for the cardinality of A. The Deng entropy is similar to the classical Shannon entropy, and it benefits from the Shannon entropy. Moreover, the belief of each focal element A is divided by a term $2^{\left|A\right|}-1$, which represents the potential number of states in A. The empty set is not included. Additionally, the Deng entropy is actually a type of composite measures, shown as follows:*

(10) $$E_{d}(m)=\;\sum_{A\subseteq X}m(A)\mathrm{lo}g_{2}(2^{\left|A\right|}-1)-m(A)\mathrm{lo}g_{2}m(A)$$

*where the term $\sum_{A\subseteq X}m(A)log_{2}(2^{\left|A\right|}-1)$ can be treated as a measure of a total nonspecficity in the mass function, and the term $-\sum_{A\subseteq X}m(A)\mathrm{lo}g_{2}m(A)$ is the measure of discord of the mass function among various focal elements.*

### 2.4. The Negation of BPA

The issue of negation has garnered significant interest since Zadeh first formally discussed the negation of deterministic probabilistic events. Smets [57] argued that the implacability and commonality function can be utilized to define the negation of the mass function $\bar{m}$. Furthermore, the equation $\bar{m}(A)=m(\bar{A})$ holds. This model is of great significance since the belief of each focal element can influence the negation of other focal elements, while there are limitations when $m(\bar{A})$ is always equal to 0 [54]. In addition, the DST offers a more general framework than the Bayes structure, and it is easier to obtain the BPA than the probability distribution in reality.

**Definition** **7.** *Yin et al. [54] proposed a method to calculate the negation of the BPA where the number of focal elements is taken into consideration. For each focal element A in the FOD, the initial belief assignment $p_{i}$ can be replaced with $1-p_{i}$. Then, calculate the sum of $\bar{m}(A)$ of all the focal elements and normalize it. Consequently, the general formula of the negation of the mass function can be derived as*

(11) $$\bar{m}(A)=\frac{1-m(A)}{n-1}$$

*where n is the number of focal elements, m is the belief of the focal elements of the initial mass function, and $\bar{m}(A)$ represents the negation of m(A).*

## 3. Measuring Negation Information in FMEA with the Belief Entropy

**Figure 1:**
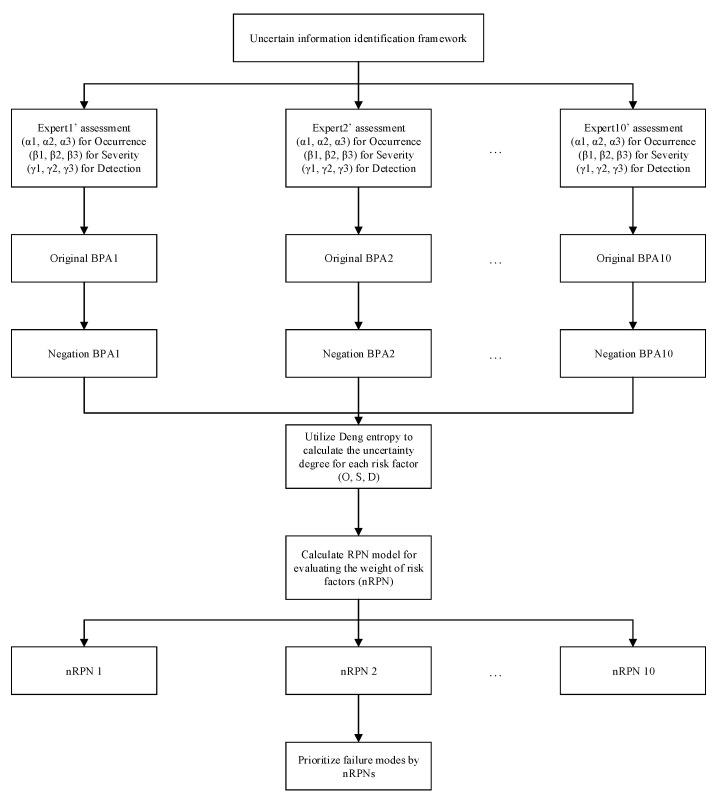
The flowchart of measuring the negation information in FMEA with the belief entropy in the DST framework.

The FMEA approach prioritizes risk items by utilizing the classical RPN value. It is used to take preventive actions against each risk item in practical settings. However, the relative importance of corresponding risk factors O,S, and *D* might be obscured within experts’ assessments. In the framework of DST, in order to reasonably deal with the relative importance of each risk factor in the FMEA method, the Deng entropy is adopted to measure the uncertainty of the negation information of the risk analysis in each risk factor. The flowchart for measuring negation information using the belief entropy within the DST framework is shown in Figure 1. The main steps of the new RPN-based FMEA method are presented as follows.

Step 1. Define the uncertain information identification framework, which is used to divide the system into subsystems and describe the operation process of the system clearly.

Step 2. FMEA experts evaluate the level and probability of each risk factor (O, S, and D).

Step 3. In the framework of evidence theory, the collected subjective evaluation from FMEA experts is modeled as a mass function, which is defined as the original BPAs.

Step 4. Calculate the negation of the original BPAs.

Step 5. Measure the degree of uncertainty of each risk factor by the Deng entropy based on the calculation result of negation BPAs.

The Deng entropy is adopted to calculate the uncertainty degree of evidence based on the negation BPAs ($\bar{m}(A)$):

(12) $$E_{d}{}^{\prime }(\bar{m})=\;-\;\sum_{A\subseteq X}\bar{m}(A)\mathrm{lo}g_{2}\frac{\bar{m}(A)}{2^{\left|A\right|}-1}$$

where the term $\bar{m}$ obtains the degree of uncertainty of the original information *m*, while *m* is the mass function defined in the frame of discernment *X*.

According to the definition of the Deng entropy, the degree of uncertainty of each risk factor for the *i*th expert can be calculated as follows:

(13) $$\begin{array}{c} E_{d}(O_{i})=\;-\;\sum_{O_{i}\in A\subseteq X}m(A)\mathrm{lo}g_{2}\frac{m(A)}{2^{\left|A\right|}-1}\\ E_{d}(S_{i})=\;-\;\sum_{S_{i}\in A\subseteq X}m(A)\mathrm{lo}g_{2}\frac{m(A)}{2^{\left|A\right|}-1}\\ E_{d}(D_{i})=\;-\;\sum_{D_{i}\in A\subseteq X}m(A)\mathrm{lo}g_{2}\frac{m(A)}{2^{\left|A\right|}-1} \end{array}$$

where *m* is the mass function defined in the frame of discernment *X*, and X={O,S,D}. Then, the formula for calculating the integrated value of the assessment results for each risk factor, $O_{i}$, $S_{i}$, and $D_{i}$, is as follows:

(14) $$\begin{array}{c} O_{i}=\;\sum_{j=1}^{10}R_{j}m_{j}\left(O_{i}\right)\\ S_{i}=\;\sum_{j=1}^{10}R_{j}m_{j}(S_{i})\\ D_{i}=\;\sum_{j=1}^{10}R_{j}m_{j}\left(D_{i}\right) \end{array}$$

where j=(1,2,3,4,5,6,7,8,9,10); $R_{1}=1,\;R_{2}=2,\;R_{3}=3,\ldots \ldots ,R_{10}=10$; and $R_{j}$ represents the rating value of the experts. $m_{j}(O_{i}),m_{j}(S_{i})$, and $m_{j}(D_{i})$ are the mass functions of the corresponding rating values evaluated by the *i*th expert.

Using the negation of the BPAs takes the place of the mass functions in Equation (14); thus, the integrated rating value of experts can be calculated as follows:

(15) $$\begin{array}{c} O_{i}{}^{\prime }=\;\sum_{j=1}^{10}R_{j}{\bar{m}}_{j}\left(O_{i}\right)\\ S_{i}{}^{\prime }=\;\sum_{j=1}^{10}R_{j}{\bar{m}}_{j}(S_{i})\\ D_{i}{}^{\prime }=\;\sum_{j=1}^{10}R_{j}{\bar{m}}_{j}\left(D_{i}\right) \end{array}$$

where $\;{\bar{m}}_{j}(O_{i})$, ${\bar{m}}_{j}(S_{i})$ and ${\bar{m}}_{j}(D_{i})$ are the negations of mass functions of the corresponding rating values evaluated by the *i*th expert.

According to Equation (13), Equation (15) can be transformed as:

(16) $$\begin{array}{c} E_{d}^{{}^{\prime }}(O_{i})=\;-\;\sum_{O_{i}\in A\subseteq X}\bar{m}(A)\mathrm{lo}g_{2}\frac{\bar{m}(A)}{2^{\left|A\right|}-1}\\ E_{d}^{{}^{\prime }}(S_{i})=\;-\;\sum_{S_{i}\in A\subseteq X}\bar{m}(A)\mathrm{lo}g_{2}\frac{\bar{m}(A)}{2^{\left|A\right|}-1}\\ E_{d}^{{}^{\prime }}(D_{i})=\;-\;\sum_{D_{i}\in A\subseteq X}\bar{m}(A)\mathrm{lo}g_{2}\frac{\bar{m}(A)}{2^{\left|A\right|}-1} \end{array}$$

where $\bar{m}$ is the negation of the original mass function *m*, which offers more valuable information beneficial for uncertain information modeling and processing in evidence theory.

Step 6. Calculate the improved RPNs for each failure mode based on the Deng entropy.

Assume that each expert in the FMEA team has the same weight in the final evaluation result. The negation BPA-based risk priority number (nRPN) with the Deng entropy for an uncertainty measure is defined as follows:

(17) $$nRPN=\;\sum_{i=1}^{n}\frac{1}{n}O_{i}^{{}^{\prime }-e^{E_{d}^{{}^{\prime }}(O_{i})}}\times S_{i}^{{}^{\prime }-e^{E_{d}^{{}^{\prime }}(S_{i})}}\times D_{i}^{{}^{\prime }-e^{E_{d}^{{}^{\prime }}(D_{i})}}$$

where $E_{d}^{{}^{\prime }}(\cdot )$ is based on the negation of the original BPA and $E_{d}^{{}^{\prime }}(\cdot )$ measures the degree of uncertainty of the experts’ assessment on the corresponding risk factor. Here, $e^{E_{d}^{{}^{\prime }}(\cdot )}$ expresses the relative weight of each risk factor based on the negation of the degree of uncertainty. Furthermore, $O_{i}$, $S_{i}$, and $D_{i}$ indicate the aggregate value of the evaluation results, which are shown by the structure of belief function for the risk factors (*O*, *S*, and *D*) assessed by the *i*th expert.

Step 7. Utilize nRPN to rank the failure modes.

According to the ranking results determined by nRPN, the priorities for potential failure modes are obtained. Thus, risk prevention and a remedial action plan can be determined by assigning finite resources to the failure mode with the highest priority.

## 4. Application and Discussion

In this section, the improved FMEA method based on the Deng entropy and negation of BPA is applied to the case of an aircraft turbine rotor blade. The effectiveness of this improved method is verified based on the case study adopted from [49,58,59].

### 4.1. Application

The case study adopted from [49,58,59] included 17 failure modes of rotor blades for an aircraft engine assessed by three FMEA experts. The 17 FMEA items were denoted as FM1, FM2, ..., FM17 for the three risk factors O, S, and D. Three FMEA experts, named Expert1, Expert2, Expert3, carried out a risk analysis. Meanwhile, three experts provided an assessment on each failure factor of these 17 failure modes based on three evaluation grades, “good”, “moderate”, and “poor”. The assessment results of the occurrence, severity, and detection of each failure mode in [58] are regarded as the original BPAs. After calculating the negation of the original BPAs for the assessments, the results are shown in Table 1.

On the basis of Equation (11), the calculation process is as follows:

For $O_{1}:$$\bar{m}(3)=\frac{1-m(3)}{n-1}=\;\frac{1-0.4}{2-1}=0.6,$$\bar{m}(4)=0.4$

For $S_{1}:$$\bar{m}(6)=\frac{1-m(6)}{n-1}=\;\frac{1-0.1}{3-1}=0.45,\bar{m}(7)=0.1,\bar{m}(8)=0.45$

For $D_{1}:$$\bar{m}(1)=\frac{1-m(1)}{n-1}=\;\frac{1-0.1}{3-1}=0.45,\bar{m}(2)=0.1,\bar{m}(3)=0.45$

Take the first FMEA item FM1 as an example. The negation of the uncertainty degree of each risk factor can be measured as follows:

$$\begin{array}{c} \begin{array}{cc} E_{d}^{{}^{\prime }}(O_{1}) & =\;-\;\sum_{O_{i}\in A\subseteq X}\bar{m}(A)\mathrm{lo}g_{2}\frac{\bar{m}(A)}{2^{\left|A\right|}-1}\\ \; & =\;-0.6\log_{2}\frac{0.6}{2^{1}-1}-0.4\log_{2}\frac{0.4}{2^{1}-1}=0.9710\\ E_{d}^{{}^{\prime }}(S_{1}) & =\;-\;\sum_{S_{i}\in A\subseteq X}\bar{m}(A)\mathrm{lo}g_{2}\frac{\bar{m}(A)}{2^{\left|A\right|}-1}=\;1.3690\\ E_{d}^{{}^{\prime }}(D_{1}) & =\;-\;\sum_{D_{i}\in A\subseteq X}\bar{m}(A)\mathrm{lo}g_{2}\frac{\bar{m}(A)}{2^{\left|A\right|}-1}=1.3690 \end{array} \end{array}$$

The aggregate value of the first expert’s assessment on each risk factor is calculated by Equation (15), which is as follows:

$$\begin{array}{c} O_{1}{}^{\prime }=\;\sum_{j=1}^{10}R_{j}{\bar{m}}_{j}\left(O_{1}\right)=\;R_{3}{\bar{m}}_{3}(O_{1})+\;R_{4}{\bar{m}}_{4}(O_{1})=\;3.4000,\\ S_{1}^{{}^{\prime }}=\;\sum_{j=1}^{10}R_{j}{\bar{m}}_{j}(S_{1})=7.0000,\\ D_{1}{}^{\prime }=\;\sum_{j=1}^{10}R_{j}{\bar{m}}_{j}\left(D_{1}\right)=2.0000 \end{array}$$

Subsequently, the degree of uncertainty of each risk factor for all three experts in FM1 can be calculated with the Deng entropy. The calculation result will be employed to calculate the weight of the risk factor. Additionally, the aggregated value of the three experts’ assessment outcomes for each risk factor can be calculated. The detailed results for FM1 are displayed in Table 2.

Calculate the nRPN based on its definition in Equation (17) for FM1:

(18) $$nRPN=\;\sum_{i=1}^{3}\frac{1}{3}O_{i}^{{}^{\prime }e^{-E_{d}^{{}^{\prime }}(O_{i})}}\times S_{i}^{{}^{\prime }e^{-E_{d}^{{}^{\prime }}(S_{i})}}\times D_{i}^{{}^{\prime }e^{-E_{d}^{{}^{\prime }}(D_{i})}}=3.8127$$

The calculation process of the nRPN method can be applied to the analysis of the other 16 potential failure modes and effects in reference [60]. The analysis and calculation results are shown in Table 3.

According to the example of reference [58], the risk items FM1, FM2, FM3, FM4, FM5, FM6, FM7, and FM8 are for the failure mode and effect analysis of compressor rotor blades, while risk items FM9, FM10, FM11, FM12, FM13, FM14, FM15, FM16, and FM17 are for turbine rotor blades. The priority of the compressor rotor blade risk items can be ranked as: FM1>FM2>FM8>FM6>FM4>FM3>FM7>FM5, while the ranking results of turbine rotor blade risk items are: FM9>FM17>FM15>FM12>FM14>FM13>FM10>FM11>FM16, where the symbol “>” indicates a higher priority. These failure modes are prioritized based on the ranking of the nRPNs. In the failure mode and effect analysis of compressor rotor blades, the RPN value of FM1 is the highest, which indicated that it is the riskiest out of eight FMs. Similarly, FM9, with the highest RPN value, is the riskiest among the turbine rotor blade risk items. The results indicate that we should pay more attention to FM1 and FM9.

### 4.2. Discussion

**Figure 2:**
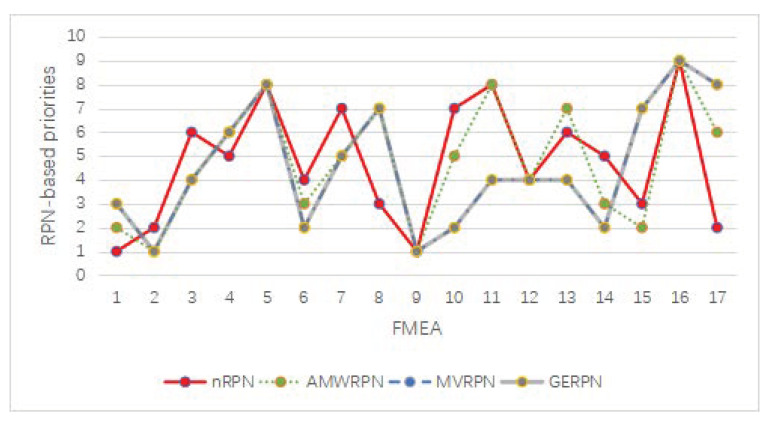
Comparative analysis of RPN ranking results based on different improved FMEA methods.

In order to verify the effectiveness of the proposed nRPN method, the results were compared with the evaluation results of ambiguity-measure-based RPN (AMRPN) [59], mean-value-based RPN (MVRPN) [58], and generalized-evidence-based RPN (GERPN) [49], where the results of these methods are rational. The analysis and calculation results are shown in Table 4 and Figure 2.

By observing the comparison results of various methods in Table 4 and Figure 2, it can be found that the mean-value-based RPN (MVRPN)and generalized-evidence-based RPN (GERPN) show repeated risk sequence values in some risk items, such as FM10 and FM14. The occurrence of duplicate values is detrimental to the selection of prevention and improvement measures for the risk item. The sorting results illustrate that the proposed method and the AMPRN method are rational or more effective without the same sorting items.

Moreover, it can be seen that the minimum ranking value belongs to FM5 in the first eight FMEA items on compressor rotor blades, which is consistent with the AMRPN method, the MVRPN method, and the GERPN method. However, FM2 no longer has the highest priority since FM1 replaces it. For FM1, it is obvious that the assessments of FMEA experts in [58] have greater uncertainty than FM2, which leads to an increase in the risk level. With regard to turbine rotor blade, the largest and smallest values measured by nRPN in the remaining nine FMEA items are FM9 and FM16, respectively, which are the same as in the methods of AMRPN, MVRPN, and GERPN. Therefore, the proposed method is rational, and it can better capture the uncertainty of FMEA experts’ evaluation.

There are also some differences that can be seen upon comparison. The risk levels of FM4 and FM8 increase in the FMEA items of compressor rotor blades. The FMEA items FM3, FM6, and FM7 are assigned lower risk priorities. This is reasonable because the tendency of different experts on FM3, FM6, and FM7 is consistent with that of [58], which means that the risk of failure mode is under the control of experts. On the contrary, the difference of experts’ assessments in FM4 and FM8 lead to a higher priority. Thus, these failure modes should be given more attention in practical work. On the other hand, it is noteworthy that the risk level of FM17 shows a dramatic increase, and FM10 is assigned lower priority compared with other methods. The uncertainty of experts’ assessments shows the same effect on the ranking results in the item of the turbine rotor blade.

What distinguishes nRPN from the aforementioned methods is that the corresponding weights of risk factors are emphasized, and more uncertain information is offered by the negation. Furthermore, the Deng entropy is adopted for the uncertainty measure in the DST, thus ensuring the internal coordination. Actually, it can distinguish the value of risk order well, which aids in selecting appropriate risk prevention measures and overcomes the limitations of the traditional RPN. In further work, the relative weights of experts and a simplification of BPA generation can be taken into consideration.

## 5. Conclusions

In this paper, a novel RPN model for the FMEA approach named nRPN is proposed to overcome some existing disadvantages in traditional RPNs. The nRPN method models the potential priority judgement of each risk factor in FMEA as an exponential weighting factor. In the framework of DST, the degree of uncertainty of subjective evaluations are transformed into the relative importance of risk factors by using the Deng entropy and negation information in the nRPN method. The characteristics and innovations of this proposed method can be summarized as follows. First, the negation of BPA is used to obtain more available uncertain information, as it is vital for providing more available information for risk analysis and decision making. Second, the Deng entropy is utilized to calculate the degree of uncertainty of negation BPAs. A case study of aircraft turbine rotor blades verifies the practicability and efficiency of the new nRPN model. It should be noted that, although the proposed method in this paper improves some shortcomings of traditional RPN and is applied to practical engineering problems, the current research does not consider other potential risk factors apart from the O, S, and D. In some cases, these are more risk factors that should be handled with caution. Moreover, the relative weight of experts should also be considered in further studies.

## Figures and Tables

**Table 1:** The negation of mass functions of 17 potential failure modes.

FMs	Risk Factor Assessment Results—Negation BPA								
	Expert1			Expert2			Expert3		
	O	S	D	O	S	D	O	S	D
1	$\bar{m}$(3) = 0.6	$\bar{m}$(6) = 0.45	$\bar{m}$(1) = 0.45	$\bar{m}$(3) = 0.1	$\bar{m}$(6) = 0.45	$\bar{m}$(1) = 0.45	$\bar{m}$(3) = 0.2	$\bar{m}$(6) = 0.45	$\bar{m}$(1) = 0.45
	$\bar{m}$(4) = 0.4	$\bar{m}$(7) = 0.1	$\bar{m}$(2) = 0.1	$\bar{m}$(4) = 0.9	$\bar{m}$(7) = 0.1	$\bar{m}$(2) = 0.1	$\bar{m}$(4) = 0.8	$\bar{m}$(7) = 0.1	$\bar{m}$(2) = 0.1
		$\bar{m}$(8) = 0.45	$\bar{m}$(3) = 0.45		$\bar{m}$(8) = 0.45	$\bar{m}$(3) = 0.45		$\bar{m}$(8) = 0.45	$\bar{m}$(3) = 0.45
2	$\bar{m}$(1) = 0.45	$\bar{m}$(7) = 0.45	$\bar{m}$(3) = 0.45	$\bar{m}$(1) = 0.45	$\bar{m}$(8) = 0.3	$\bar{m}$(3) = 0.45	$\bar{m}$(1) = 0.45	$\bar{m}$(7) = 0.45	$\bar{m}$(3) = 0.45
	$\bar{m}$(2) = 0.1	$\bar{m}$(8) = 0.1	$\bar{m}$(4) = 0.1	$\bar{m}$(2) = 0.1	$\bar{m}$(9) = 0.7	$\bar{m}$(4) = 0.1	$\bar{m}$(2) = 0.1	$\bar{m}$(8) = 0.1	$\bar{m}$(4) = 0.1
	$\bar{m}$(3) = 0.45	$\bar{m}$(9) = 0.45	$\bar{m}$(5) = 0.45	$\bar{m}$(3) = 0.45		$\bar{m}$(5) = 0.45	$\bar{m}$(3) = 0.45	$\bar{m}$(9) = 0.45	$\bar{m}$(5) = 0.45
3	$\bar{m}$(0) = 0.45	$\bar{m}$(9) = 0.45	$\bar{m}$(2) = 0.45	$\bar{m}$(0) = 0.45	$\bar{m}$(9) = 0.45	$\bar{m}$(2) = 0.45	$\bar{m}$(0) = 0.45	$\bar{m}$(9) = 0.45	$\bar{m}$(2) = 0.45
	$\bar{m}$(1) = 0.1	$\bar{m}$(10) = 0.1	$\bar{m}$(3) = 0.1	$\bar{m}$(1) = 0.1	$\bar{m}$(10) = 0.1	$\bar{m}$(3) = 0.1	$\bar{m}$(1) = 0.1	$\bar{m}$(10) = 0.1	$\bar{m}$(3) = 0.1
	$\bar{m}$(2) = 0.45	$\bar{m}$(11) = 0.45	$\bar{m}$(4) = 0.45	$\bar{m}$(2) = 0.45	$\bar{m}$(11) = 0.45	$\bar{m}$(4) = 0.45	$\bar{m}$(2) = 0.45	$\bar{m}$(11) = 0.45	$\bar{m}$(4) = 0.45
4	$\bar{m}$(0) = 0.45	$\bar{m}$(6) = 0.2	$\bar{m}$(2) = 0.45	$\bar{m}$(0) = 0.45	$\bar{m}$(5) = 0.45	$\bar{m}$(2) = 0.7	$\bar{m}$(0) = 0.45	$\bar{m}$(5) = 0.45	$\bar{m}$(2) = 0.45
	$\bar{m}$(1) = 0.1	$\bar{m}$(7) = 0.8	$\bar{m}$(3) = 0.1	$\bar{m}$(1) = 0.1	$\bar{m}$(6) = 0.1	$\bar{m}$(3) = 0.3	$\bar{m}$(1) = 0.1	$\bar{m}$(6) = 0.1	$\bar{m}$(3) = 0.1
	$\bar{m}$(2) = 0.45		$\bar{m}$(4) = 0.45	$\bar{m}$(2) = 0.45	$\bar{m}$(7) = 0.45		$\bar{m}$(2) = 0.45	$\bar{m}$(7) = 0.45	$\bar{m}$(4) = 0.45
5	$\bar{m}$(0) = 0.45	$\bar{m}$(2) = 0.45	$\bar{m}$(1) = 0.5	$\bar{m}$(0) = 0.45	$\bar{m}$(2) = 0.45	$\bar{m}$(1) = 0.3	$\bar{m}$(0) = 0.45	$\bar{m}$(2) = 0.6	$\bar{m}$(0) = 0.45
	$\bar{m}$(1) = 0.1	$\bar{m}$(3) = 0.1	$\bar{m}$(2) = 0.5	$\bar{m}$(1) = 0.1	$\bar{m}$(3) = 0.1	$\bar{m}$(2) = 0.7	$\bar{m}$(1) = 0.1	$\bar{m}$(3) = 0.4	$\bar{m}$(1) = 0.1
	$\bar{m}$(2) = 0.45	$\bar{m}$(4) = 0.45		$\bar{m}$(2) = 0.45	$\bar{m}$(4) = 0.45		$\bar{m}$(2) = 0.45		$\bar{m}$(2) = 0.45
6	$\bar{m}$(1) = 0.45	$\bar{m}$(5) = 0.45	$\bar{m}$(4) = 0.45	$\bar{m}$(1) = 0.45	$\bar{m}$(5) = 0.45	$\bar{m}$(4) = 0.45	$\bar{m}$(1) = 0.45	$\bar{m}$(5) = 0.45	$\bar{m}$(4) = 0.45
	$\bar{m}$(2) = 0.1	$\bar{m}$(6) = 0.1	$\bar{m}$(5) = 0.1	$\bar{m}$(2) = 0.1	$\bar{m}$(6) = 0.1	$\bar{m}$(5) = 0.1	$\bar{m}$(2) = 0.1	$\bar{m}$(6) = 0.1	$\bar{m}$(5) = 0.1
	$\bar{m}$(3) = 0.45	$\bar{m}$(7) = 0.45	$\bar{m}$(6) = 0.45	$\bar{m}$(3) = 0.45	$\bar{m}$(7) = 0.45	$\bar{m}$(6) = 0.45	$\bar{m}$(3) = 0.45	$\bar{m}$(7) = 0.45	$\bar{m}$(6) = 0.45
7	$\bar{m}$(0) = 0.45	$\bar{m}$(6) = 0.45	$\bar{m}$(2) = 0.45	$\bar{m}$(0) = 0.45	$\bar{m}$(6) = 0.45	$\bar{m}$(2) = 0.45	$\bar{m}$(0) = 0.45	$\bar{m}$(6) = 0.45	$\bar{m}$(2) = 0.45
	$\bar{m}$(1) = 0.1	$\bar{m}$(7) = 0.1	$\bar{m}$(3) = 0.1	$\bar{m}$(1) = 0.1	$\bar{m}$(7) = 0.1	$\bar{m}$(3) = 0.1	$\bar{m}$(1) = 0.1	$\bar{m}$(7) = 0.1	$\bar{m}$(3) = 0.1
	$\bar{m}$(2) = 0.45	$\bar{m}$(8) = 0.45	$\bar{m}$(4) = 0.45	$\bar{m}$(2) = 0.45	$\bar{m}$(8) = 0.45	$\bar{m}$(4) = 0.45	$\bar{m}$(2) = 0.45	$\bar{m}$(8) = 0.45	$\bar{m}$(4) = 0.45
8	$\bar{m}$(2) = 0.45	$\bar{m}$(5) = 0.4	$\bar{m}$(0) = 0.45	$\bar{m}$(2) = 0.45	$\bar{m}$(5) = 0.2	$\bar{m}$(0) = 0.45	$\bar{m}$(2) = 0.45	$\bar{m}$(5) = 0.2	$\bar{m}$(0) = 0.45
	$\bar{m}$(3) = 0.1	$\bar{m}$(6) = 0.6	$\bar{m}$(1) = 0.1	$\bar{m}$(3) = 0.1	$\bar{m}$(6) = 0.8	$\bar{m}$(1) = 0.1	$\bar{m}$(3) = 0.1	$\bar{m}$(7) = 0.8	$\bar{m}$(1) = 0.1
	$\bar{m}$(4) = 0.45		$\bar{m}$(2) = 0.45	$\bar{m}$(4) = 0.45		$\bar{m}$(2) = 0.45	$\bar{m}$(4) = 0.45		$\bar{m}$(2) = 0.45
9	$\bar{m}$(1) = 0.9	$\bar{m}$(9) = 0.6	$\bar{m}$(3) = 0.45	$\bar{m}$(1) = 0.75	$\bar{m}$(9) = 0.9	$\bar{m}$(3) = 0.45	$\bar{m}$(1) = 0.8	$\bar{m}$(9) = 0.9	$\bar{m}$(3) = 0.45
	$\bar{m}$(2) = 0.1	$\bar{m}$(10) = 0.4	$\bar{m}$(4) = 0.1	$\bar{m}$(2) = 0.25	$\bar{m}$(10) = 0.1	$\bar{m}$(4) = 0.1	$\bar{m}$(2) = 0.2	$\bar{m}$(10) = 0.1	$\bar{m}$(4) = 0.1
			(5) = 0.45			$\bar{m}$(5) = 0.45			$\bar{m}$(5) = 0.45
10	$\bar{m}$(0) = 0.45	$\bar{m}$(9) = 0.45	$\bar{m}$(5) = 0.45	$\bar{m}$(0) = 0.45	$\bar{m}$(9) = 0.45	$\bar{m}$(5) = 0.45	$\bar{m}$(0) = 0.45	$\bar{m}$(9) = 0.45	$\bar{m}$(5) = 0.45
	$\bar{m}$(1) = 0.1	$\bar{m}$(10) = 0.1	$\bar{m}$(6) = 0.1	$\bar{m}$(1) = 0.1	$\bar{m}$(10) = 0.1	$\bar{m}$(6) = 0.1	$\bar{m}$(1) = 0.1	$\bar{m}$(10) = 0.1	$\bar{m}$(6) = 0.1
	$\bar{m}$(2) = 0.45	$\bar{m}$(11) = 0.45	$\bar{m}$(7) = 0.45	$\bar{m}$(2) = 0.45	$\bar{m}$(11) = 0.45	$\bar{m}$(7) = 0.45	$\bar{m}$(2) = 0.45	$\bar{m}$(11) = 0.45	$\bar{m}$(7) = 0.45
11	$\bar{m}$(0) = 0.45	$\bar{m}$(9) = 0.45	v(4) = 0.45	(0) = 0.45	$\bar{m}$(9) = 0.45	$\bar{m}$(4) = 0.45	$\bar{m}$(0) = 0.45	$\bar{m}$(9) = 0.45	$\bar{m}$(4) = 0.45
	$\bar{m}$(1) = 0.1	$\bar{m}$(10) = 0.1	$\bar{m}$(5) = 0.1	$\bar{m}$(1) = 0.1	$\bar{m}$(10) = 0.1	$\bar{m}$(5) = 0.1	$\bar{m}$(1) = 0.1	$\bar{m}$(10) = 0.1	$\bar{m}$(5) = 0.1
	$\bar{m}$(2) = 0.45	$\bar{m}$(11) = 0.45	$\bar{m}$(6) = 0.45	$\bar{m}$(2) = 0.45	$\bar{m}$(11) = 0.45	$\bar{m}$(6) = 0.45	$\bar{m}$(2) = 0.45	$\bar{m}$(11) = 0.45	$\bar{m}$(6) = 0.45
12	$\bar{m}$(0) = 0.45	$\bar{m}$(9) = 0.45	$\bar{m}$(5) = 0.6	$\bar{m}$(0) = 0.45	$\bar{m}$(9) = 0.45	$\bar{m}$(4) = 0.8	$\bar{m}$(0) = 0.45	$\bar{m}$(9) = 0.45	$\bar{m}$(5) = 0.7
	$\bar{m}$(1) = 0.1	$\bar{m}$(10) = 0.1	$\bar{m}$(6) = 0.4	$\bar{m}$(1) = 0.1	$\bar{m}$(10) = 0.1	$\bar{m}$(5) = 0.2	$\bar{m}$(1) = 0.1	$\bar{m}$(10) = 0.1	$\bar{m}$(6) = 0.3
	$\bar{m}$(2) = 0.45	$\bar{m}$(11) = 0.45		$\bar{m}$(2) = 0.45	$\bar{m}$(11) = 0.45		$\bar{m}$(2) = 0.45	$\bar{m}$(11) = 0.45	
13	$\bar{m}$(0) = 0.45	$\bar{m}$(9) = 0.45	$\bar{m}$(4) = 0.8	$\bar{m}$(0) = 0.45	$\bar{m}$(9) = 0.45	$\bar{m}$(4) = 0.45	$\bar{m}$(0) = 0.45	$\bar{m}$(9) = 0.45	$\bar{m}$(4) = 0.45
	$\bar{m}$(1) = 0.1	$\bar{m}$(10) = 0.1	$\bar{m}$(5) = 0.2	$\bar{m}$(1) = 0.1	$\bar{m}$(10) = 0.1	$\bar{m}$(5) = 0.1	$\bar{m}$(1) = 0.1	$\bar{m}$(10) = 0.1	$\bar{m}$(5) = 0.1
	$\bar{m}$(2) = 0.45	$\bar{m}$(11) = 0.45		$\bar{m}$(2) = 0.45	$\bar{m}$(11) = 0.45	$\bar{m}$(6) = 0.45	$\bar{m}$(2) = 0.45	$\bar{m}$(11) = 0.45	$\bar{m}$(6) = 0.45
14	$\bar{m}$(0) = 0.45	$\bar{m}$(9) = 0.45	$\bar{m}$(5) = 0.45	$\bar{m}$(0) = 0.45	$\bar{m}$(9) = 0.45	$\bar{m}$(6) = 0.2	$\bar{m}$(0) = 0.45	$\bar{m}$(9) = 0.45	$\bar{m}$(5) = 0.45
	$\bar{m}$(1) = 0.1	$\bar{m}$(10) = 0.1	$\bar{m}$(6) = 0.1	$\bar{m}$(1) = 0.1	$\bar{m}$(10) = 0.1	$\bar{m}$(7) = 0.8	$\bar{m}$(1) = 0.1	$\bar{m}$(10) = 0.1	$\bar{m}$(6) = 0.1
	$\bar{m}$(2) = 0.45	$\bar{m}$(11) = 0.45	$\bar{m}$(7) = 0.45	$\bar{m}$(2) = 0.45	$\bar{m}$(11) = 0.45		$\bar{m}$(2) = 0.45	$\bar{m}$(11) = 0.45	$\bar{m}$(7) = 0.45
15	$\bar{m}$(1) = 0.45	$\bar{m}$(6) = 0.95	$\bar{m}$(2) = 0.45	$\bar{m}$(1) = 0.45	$\bar{m}$(6) = 0.45	$\bar{m}$(2) = 0.45	$\bar{m}$(1) = 0.45	$\bar{m}$(6) = 0.45	$\bar{m}$(3) = 0.3
	$\bar{m}$(2) = 0.1	$\bar{m}$(7) = 0.05	$\bar{m}$(3) = 0.1	$\bar{m}$(2) = 0.1	$\bar{m}$(7) = 0.1	$\bar{m}$(3) = 0.1	$\bar{m}$(2) = 0.1	$\bar{m}$(7) = 0.1	$\bar{m}$(4) = 0.7
	$\bar{m}$(3) = 0.45		$\bar{m}$(4) = 0.45	$\bar{m}$(3) = 0.45	$\bar{m}$(8) = 0.45	$\bar{m}$(4) = 0.45	$\bar{m}$(3) = 0.45	$\bar{m}$(8) = 0.45	
16	$\bar{m}$(1) = 0.9	$\bar{m}$(3) = 0.45	$\bar{m}$(2) = 0.45	$\bar{m}$(1) = 0.75	$\bar{m}$(3) = 0.45	$\bar{m}$(2) = 0.45	$\bar{m}$(1) = 0.8	$\bar{m}$(3) = 0.45	$\bar{m}$(2) = 0.8
	$\bar{m}$(2) = 0.1	$\bar{m}$(4) = 0.1	$\bar{m}$(3) = 0.1	$\bar{m}$(2) = 0.25	$\bar{m}$(4) = 0.1	$\bar{m}$(3) = 0.1	$\bar{m}$(2) = 0.2	$\bar{m}$(4) = 0.1	$\bar{m}$(3) = 0.2
		$\bar{m}$(5) = 0.45	$\bar{m}$(4) = 0.45		$\bar{m}$(5) = 0.45	$\bar{m}$(4) = 0.45		$\bar{m}$(5) = 0.45	
17	$\bar{m}$(1) = 0.45	$\bar{m}$(5) = 0.1	$\bar{m}$(2) = 0.45	$\bar{m}$(1) = 0.45	$\bar{m}$(5) = 0.1	$\bar{m}$(2) = 0.45	$\bar{m}$(1) = 0.45	$\bar{m}$(5) = 0.4	$\bar{m}$(2) = 0.45
	$\bar{m}$(2) = 0.1	$\bar{m}$(6) = 0.9	$\bar{m}$(3) = 0.1	$\bar{m}$(2) = 0.1	$\bar{m}$(6) = 0.9	$\bar{m}$(3) = 0.1	$\bar{m}$(2) = 0.1	$\bar{m}$(6) = 0.6	$\bar{m}$(3) = 0.1
	$\bar{m}$(3) = 0.45		$\bar{m}$(4) = 0.45	$\bar{m}$(3) = 0.45		$\bar{m}$(4) = 0.45	$\bar{m}$(3) = 0.45		$\bar{m}$(4) = 0.45

**Table 2:** The uncertainty degree and assessment results of each risk factor for the experts.

FM1	Expert1	Expert2	Expert3
	${E_{d}}^{\prime }(O_{1})$ = 0.9710	${E_{d}}^{\prime }(O_{2})$ = 0.4690	${E_{d}}^{\prime }(O_{3})$ = 0.7219
	${E_{d}}^{\prime }(S_{1})$ = 1.3690	${E_{d}}^{\prime }(S_{2})$ = 1.3690	${E_{d}}^{\prime }(S_{3})$ = 1.3690
	${E_{d}}^{\prime }(D_{1})$ = 1.3690	${E_{d}}^{\prime }(D_{2})$ = 1.3690	${E_{d}}^{\prime }(D_{3})$ = 1.3690
Rating	${O_{1}}^{\prime }=3.4000$	${O_{2}}^{\prime }=3.9000$	${O_{3}}^{\prime }=3.8000$
	${S_{1}}^{\prime }=7.0000$	${S_{2}}^{\prime }=7.0000$	${S_{3}}^{\prime }=7.0000$
	${D_{1}}^{\prime }=2.0000$	${D_{2}}^{\prime }=2.0000$	${D_{3}}^{\prime }=2.0000$

**Table 3:** The nRPN values and ranking of risk items.

Component	Compressor Rotor Blades								Turbo Rotor Blades								
Failure mode	1	2	3	4	5	6	7	8	9	10	11	12	13	14	15	16	17
nRPN	3.81	3.31	2.38	2.56	1.53	2.83	2.17	2.98	5.32	2.83	2.70	3.5	3.01	3.41	4.02	2.12	4.20
Rank	1	2	6	5	8	4	7	3	1	7	8	4	6	5	3	9	2

**Table 4:** The ranking of failure modes based on the proposed method in comparison with existing methods.

FM	nRPN	Rank	AMRPN [59]	Rank	MVRPN [58]	Rank	GERPN [49]	Rank
1	3.8127	1	5.1551	2	42.56	3	3.491	3
2	3.3062	2	5.3174	1	64	1	3.9994	1
3	2.3753	6	3.8684	4	30	4	3.1069	4
4	2.5564	5	3.3302	6	18	6	2.6205	6
5	1.5252	8	1.6529	8	4.17	8	1.6095	8
6	2.8333	4	5.0964	3	60	2	3.9143	2
7	2.1693	7	3.3567	5	21	5	2.7586	5
8	2.9844	3	3.2975	7	15	7	2.466	7
9	5.3239	1	8.3797	1	78.92	1	4.2881	1
10	2.8333	7	5.0964	5	60	2	3.9143	2
11	2.7049	8	4.7399	8	50	4	3.6836	4
12	3.531	4	5.0973	4	50	4	3.6836	4
13	3.0056	6	4.9447	7	50	4	3.6836	4
14	3.4084	5	5.4187	3	60	2	3.9143	2
15	4.0158	3	5.9509	2	42	7	3.4756	7
16	2.1183	9	3.756	9	23.88	9	2.8794	9
17	4.2019	2	5.0554	6	30.05	8	3.1089	8

## Data Availability

All data generated or analysed during this study are included in this published article.
